# An Unusual Case of Buerger’s Disease in a Pregnant Female

**DOI:** 10.7759/cureus.28382

**Published:** 2022-08-25

**Authors:** Zalak V Karena, Aditya D Mehta, Rajvee Rao, Nandan Gowda, Vishnu A Gadhia

**Affiliations:** 1 Department of Obstetrics and Gynecology, Pandit Deendayal Upadhyay Medical College, Rajkot, IND; 2 Department of Radiology, Pandit Deendayal Upadhyay Medical College, Rajkot, IND

**Keywords:** caesarean section, doppler ultrasonography, thromboangiitis obliterans, pregnancy, dry gangrene, buerger’s disease

## Abstract

Thromboangitis obliterans or Buerger's disease is a segmental inflammatory condition of small and medium-sized arteries and veins. It is commonly seen in males with age under 45 years and with a current or recent history of tobacco use, and in smokers. It is sporadic in young women. This report describes a primigravida with dry gangrene in both upper and lower limbs because of Buerger’s disease. The primary diagnosis of the disease occurred first time in pregnancy at the 17th week of gestation with the patient reporting dry gangrene and pain in the digits and confirmed with a non-invasive Doppler study. The patient was screened for autoimmune diseases, diabetes mellitus, and the presence of hypercoagulable disorders. Echocardiography and arteriography were performed to rule out any source of emboli. The case report aims to discuss a rare diagnostic and therapeutic dilemma in the case of a pregnant woman presenting with gangrene without any history of tobacco addiction.

## Introduction

Thromboangiitis obliterans (TAO) or Buerger’s disease is a segmental inflammatory condition of small and medium-sized arteries and veins of upper and lower extremities. It is characterized by thrombosis and recanalization of the vessels. It is a non-atherosclerotic occlusive inflammatory disease of the upper and lower extremities of small and medium-sized vessels [1,2,3]. The prevalence of the disease among all patients with peripheral arterial disease ranges from values as low as 0.5 to 5.6% in Western Europe to values as high as 45% to 63% in India [4]. Although initially described in men, the condition occurs in women, who comprise 8% to 20% of patients in recent North American case series. This relative rise in incidence in women is likely due to increased cigarette smoking among women over the 20th century [5]. Though tobacco consumption is a strong risk factor for developing Buerger's disease, the basic pathophysiology of the disease is still not known. High levels of oxidative stress are detected in patients with Buerger's disease, concerning healthy smokers. Studies have shown an imbalance between oxidative biomarkers and the antioxidant defense system [6]. The theories to the pathogenesis of Buerger's disease include infections, coagulation disorders, or possibly inflammatory response to vascular endothelium injury. In many studies, periodontal infections, the resultant bacteriemia, periodontal infectious pockets, and inflamed lymph nodes have been identified [7]. Increased expression of selectin E, ICAM-1, and VCAM-1 integrin and morphological features of endothelial activation in Buerger's disease indicate inflammatory response to vascular endothelial injury [7].

The diagnostic criteria of Olin (2000) include age under 45 years; current or recent history of tobacco use; the presence of distal-extremity ischemia indicated by claudication pain at rest, ischemic ulcers, or gangrenes documented by non-invasive vascular testing; exclusion of autoimmune diseases, hypercoagulable states and diabetes mellitus, presence of arterial calcification; exclusion of a proximal source of emboli by echocardiography or arteriography; consistent arteriographic findings in the clinically involved and non-involved limbs [8]. During normal pregnancy, the hemostatic balance changes in the direction of hypercoagulability to combat the bleeding complications of delivery. These include increased tissue factor pathway inhibitor, increased endogenous thrombin generation, acquired activated protein C resistance, slightly decreased activated partial thromboplastin time (aPTT), and increased prothrombin complex level (PT) measured as an international normalized ratio (INR) of less than 0.9 [9]. Thus, Buerger’s disease worsens during pregnancy due to hypercoagulability associated with it. This report describes a primigravida with Buerger’s disease leading to dry gangrene in both upper and lower limbs. It aims to discuss a rare diagnostic and therapeutic dilemma in the case of a pregnant woman presenting with gangrene without any history of tobacco addiction.

## Case presentation

**Figure 1:**
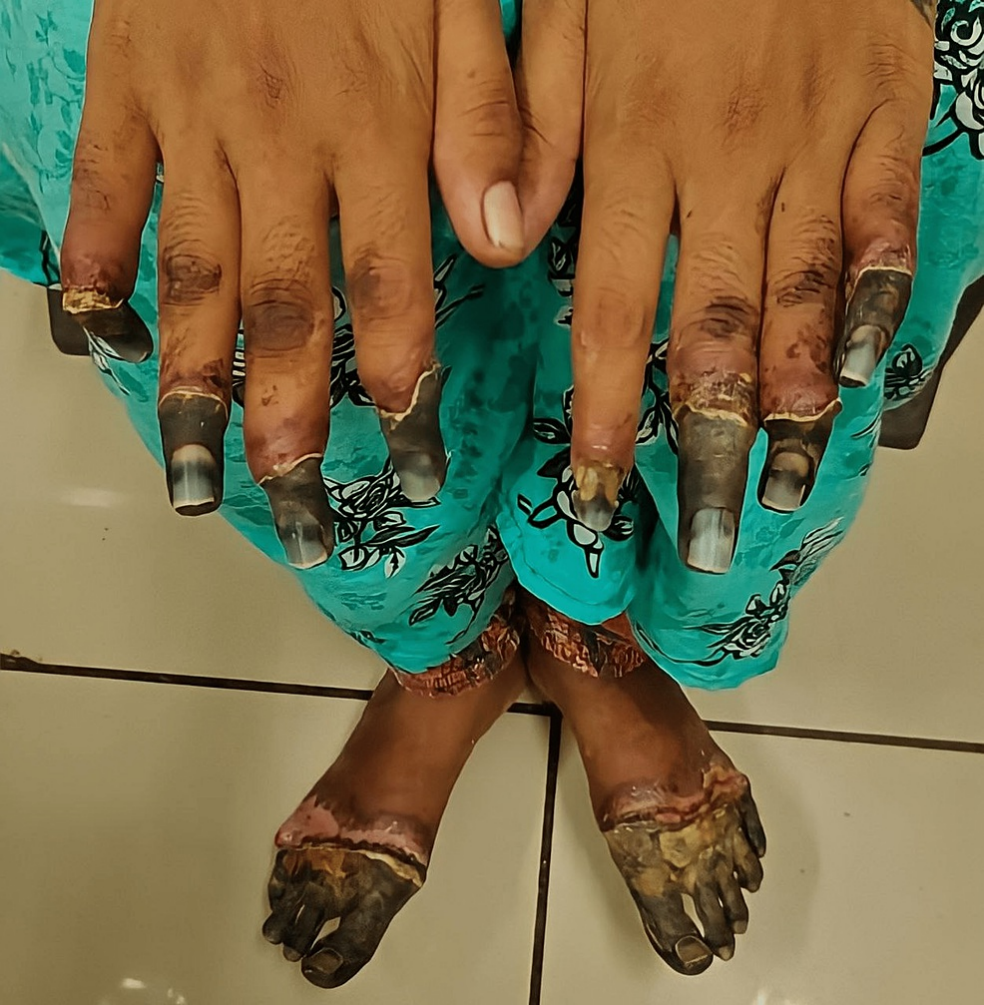
Buerger's disease with dry gangrene in pregnancy

A 22-year-old primigravida patient presented with progressive blackening of fingers of both the hands and feet bilaterally, following claudication pain for three years, preceding with non-healing ulcer and flaking skin over the lesions at the 17th week of gestation. There was no history of tobacco addiction in any form. The patient was a non-smoker and had no history of exposure to passive smoking. There was no personal or family history of cardiac events, diabetes, or hypertension. On general examination, the patient was normotensive with mild pallor. No abnormalities were detected on systemic examination. On bilateral examination of the fingers of the upper limb, dry and shriveled skin which was brown to black, having a clear line of demarcation at 2-3 cm from the terminal end of nine digits, sparing the thumb of the left hand was observed (Figure 1).

**Figure 2:**
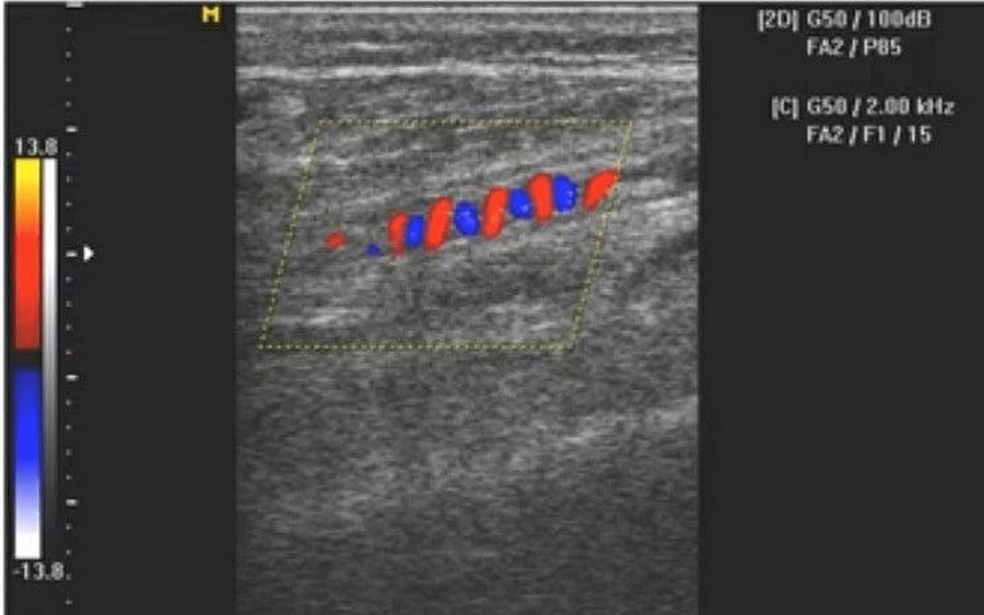
Doppler study image of lower limb showing distal small-sized corkscrew collateral vessels

The extremities of the limb were colder. Brachial artery pulsations were normal. Radial artery pulsations were regular and collapsing in nature. On bilateral examination of the toes of the lower limb, there was dry and shriveled skin which was brown to black, having a clear line of demarcation at 4-5 cm from the terminal ends of all ten digits. Dorsalis pedis arterial pulsations were not palpable. On hematological investigations, the patient’s platelet count was 4.29 lacs/cumm and hematocrit 29.8%. The rest of the blood indices were within the normal range. The patient's blood glucose levels and lipid profile were normal. Human leukocyte antigen ​​(HLA) B5 (51/52), antinuclear antibody (ANA)** **profile, tuberculosis (TB)antigen enzyme-linked immunosorbent assay (ELISA), beta-2 glycoprotein (IgG and IgM), anti-cardiolipin antibody, anti-phospholipid antibody, lupus anticoagulant testing were done to determine any genetic predisposition, antiphospholipid antibody syndrome, other autoimmune disorders, and infections which came out to be negative. Two-dimensional echocardiography was done to exclude the source of any emboli and atherosclerotic changes, and the reports pointed out no positive findings except reduced left ventricular compliance. Bilateral upper limb arterial doppler showed monophasic flow with reduced volume in both radial and ulnar arteries, whereas normal triphasic flow was noted in the axillary and brachial arteries. Bilateral lower limb arterial doppler showed biphasic flow with normal volume in the proximal right popliteal artery and anterior tibial artery and distally there were corkscrew collaterals (Figure 2).

A probable diagnosis of medium vessel vasculitis - Buerger’s disease was made. The patient was put on the treatment of tablet aspirin 75 mg, prednisolone 5 mg, azathioprine 50 mg, and nifedipine 10 mg. Exercise and arm and leg compression were advised. The patient had improvement in pain and there was a halt in the progression of gangrene size with medical treatment.

At 37 weeks of gestation, the patient developed premature rupture of membranes and was admitted to the labor room, and the pregnancy was terminated by cesarean section in case of fetal distress, with all necessary precautions. The newborn delivered had intrauterine growth restriction, and was kept under pediatric observation. The postoperative period of the patient (mother) was uneventful. The medical line of treatment for Buerger’s disease included tablet prednisolone, azathioprine, and nifedipine, which was continued postoperatively. With the termination of pregnancy, the patient was referred to a general surgeon, and at present, the patient is on medical management.

## Discussion

Buerger’s disease or thromboangiitis obliterans (TAO) though found in all ethnic groups, is seen mostly in India, Asia, and the Middle East. TAO constitute 4% to 5% of all peripheral vascular disease cases [10,11]. The usual presentation of such patients is muscular pain, termed claudication. It occurs due to diminished blood supply and is triggered by walking and activity and is relieved by rest. Rest pain is a severe form of claudication where pain persists in rest. Our patient had a burning and tingling sensation in bilateral distal upper limbs and claudication pain in bilateral lower limbs for three years which was ignored by the patient until the development of gangrene. Tobacco is the primary cause of the initiation and progression of the disease [12].

TAO is less common in females, but the incidence is increasing due to increased smoking in females. Though in our case, the HLA B5 was normal, in many cases, it is elevated, showing a genetic predisposition to the disease [13]. Studies have shown a higher prevalence of antiphospholipid antibodies, D-dimer, tissue plasminogen activator (tPA), plasminogen activator inhibitor-1​​​​​​​ (PAI-1), and fibrinopeptide suggesting disrupted coagulation [7], which were normal in our case. The basic pathophysiology of TAO: consumption of tobacco components, elevated serum anti-endothelial cell antibody titers or the infection, and the following inflammation none of the predisposing factors were identified in our case. There is the activation of platelets followed by the release of beta-thromboglobulin and platelet factor four in normal pregnancy. Blood coagulation factors except for Factor (F) XI and fibrinogen are increased in pregnancy. The level of free protein S decreases markedly. Blood coagulation inhibitors are mainly unchanged [9]. These physiological coagulatory changes during pregnancy were the only attributable factors explainable to the pathophysiology of Buerger's disease in our case.

Sympathomimetic drugs, trauma, and cold have been found to trigger TAO in non-smokers. The histopathological finding shows the intact architecture of the internal elastic lamina of the vessels involved. Except in cases of non-peculiar involvement, a biopsy is not required to diagnose TAO. Only, distal circulation of the limbs - infra-popliteal in the lower extremities and distal to the brachial artery in the upper limb - is involved [14]. Angiography typically shows corkscrew collateral vessels. In our case, we could not find any predisposing factor for Buerger’s disease in the patient except the hypercoagulable state of pregnancy.

Discontinuation of tobacco consumption and smoking has proven to be the first-line management to prevent disease progression. The gangrenous part should be protected from infections, and local hygiene should be maintained [11]. Calcium channel blockers, vasodilators, thrombolytic agents, anticoagulants, and corticosteroids are frequently recommended, with inadequate evidence [15]. Prostaglandins like iloprost and bosentan - endothelin-1 receptor antagonists have been shown to resolve distal extremity trophic changes, reducing the amputation rate [13]. Intravenous iloprost is more effective than aspirin for rest pain and healing ischemic ulcers [16]. However, oral iloprost is not effective compared to placebo. Percutaneous transluminal angioplasty is effective to restore adequate blood flow in case of limb ischemia in Buerger's disease to relieve rest pain and for limb salvage [17]. Amputation of a limb or a limb segment is done only after gangrene has set in with a clear demarcation line after smoking and tobacco cessation. 

Our patient had the development of Buerger's disease and the detection of gangrene, for the very first time during pregnancy. The newborn had intrauterine growth restriction. This is similar to the previously described eight cases of pregnancy with Buerger's disease, of which five had worsening of the disease and in one case there was intrauterine growth restriction [18].

## Conclusions

Buerger’s disease has rapid disease progression in pregnancy, and timely restraint of the disease by necessary anticoagulants and vasodilators checked the disease progression in our patient. Timely sought medical help, proper diagnosis, and prompt treatment can prevent amputation rates further. Since very few cases of Buerger's disease in females with pregnancy are available in the literature review, more such cases discussed will add to the better clinical management of such cases.

## References

[1] Fazeli B, Ligi D, Keramat S, Maniscalco R, Sharebiani H, Mannello F (2021). Recent updates and advances in Winiwarter-Buerger disease (thromboangiitis obliterans): biomolecular mechanisms, diagnostics and clinical consequences. Diagnostics (Basel).

[2] Rivera-Chavarría IJ, Brenes-Gutiérrez JD (2016). Thromboangiitis obliterans (Buerger's disease). Ann Med Surg (Lond).

[3] Igari K, Kudo T, Toyofuku T, Inoue Y (2017). Endothelial dysfunction in patients with Buerger disease. Vasc Health Risk Manag.

[4] Vijayakumar A, Tiwari R, Kumar Prabhuswamy V (2013). Thromboangiitis obliterans (Buerger's disease)-current practices. Int J Inflam.

[5] Mills JL Sr (2003). Buerger's disease in the 21st century: diagnosis, clinical features, and therapy. Semin Vasc Surg.

[6] Sharebiani H, Fazeli B, Maniscalco R, Ligi D, Mannello F (2020). The imbalance among oxidative biomarkers and antioxidant defense systems in thromboangiitis obliterans (Winiwarter-Buerger Disease). J Clin Med.

[7] Małecki R, Kluz J, Przeździecka-Dołyk J, Adamiec R (2015). The pathogenesis and diagnosis of thromboangiitis obliterans: is it still a mystery?. Adv Clin Exp Med.

[8] Olin JW (2000). Thromboangiitis obliterans (Buerger's disease). N Engl J Med.

[9] Hellgren M (2003). Hemostasis during normal pregnancy and puerperium. Semin Thromb Hemost.

[10] Lee KS, Paik CN, Chung WC (2010). Colon ischemia associated with Buerger's disease: case report and review of the literature. Gut Liver.

[11] Ansari A (1990). Thromboangiitis obliterans: current perspectives and future directions. Tex Heart Inst J.

[12] Kobayashi M, Sugimoto M, Komori K (2014). Endarteritis obliterans in the pathogenesis of Buerger's disease from the pathological and immunohistochemical points of view. Circ J.

[13] Shapouri-Moghaddam A, Mohammadi M, Rahimi HR (2019). The association of HLA-A, B and DRB1 with Buerger's disease. Rep Biochem Mol Biol.

[14] Wadud MA, Taimur SDM, Kabir CMS (2015). Buerger’s disease (thromboangiitis obliterans): a diagnostic challenge-a rare case report. BIRDEM Med J.

[15] Cacione DG, Macedo CR, Baptista-Silva JC (2016). Pharmacological treatment for Buerger's disease. Cochrane Database Syst Rev.

[16] Fiessinger JN, Schäfer M (1990). Trial of iloprost versus aspirin treatment for critical limb ischaemia of thromboangiitis obliterans. The TAO study. Lancet.

[17] Kaçmaz F, Kaya A, Yazıcı A (2016). Successful sequential drug eluting balloon angioplasty to chronic total occluded popliteal artery in a patient with thromboangitis obliterans by PCR. Anatol J Cardiol.

[18] Le Joncour A, Espitia O, Soudet S, Cacoub P, Lambert M, Saadoun D (2021). Women and pregnancy in thromboangiitis obliterans. J Vasc Res.

